# Co‐cultivation of *Thermoanaerobacter* strains with a methanogenic partner enhances glycerol conversion

**DOI:** 10.1111/1751-7915.13506

**Published:** 2020-03-10

**Authors:** Carla Pereira Magalhães, Joaquim A. Ribeiro, Ana P. Guedes, Ana L. Arantes, Diana Z. Sousa, Alfons J. M. Stams, Maria M. Alves, Ana Júlia Cavaleiro

**Affiliations:** ^1^ Centre of Biological Engineering University of Minho Braga Portugal; ^2^ Laboratory of Microbiology Wageningen University and Research Wageningen The Netherlands; ^3^ Present address: Optimizer ‐ Serviços e Consultadoria Informática Lda. Porto Portugal; ^4^ Present address: Agricultural Superior School of Ponte de Lima Polytechnic Institute of Viana do Castelo Viana do Castelo Portugal

## Abstract

Glycerol‐rich waste streams produced by the biodiesel, bioethanol and oleochemical industries can be treated and valorized by anaerobic microbial communities to produce methane. As current knowledge of the microorganisms involved in thermophilic glycerol conversion to methane is scarce, thermophilic glycerol‐degrading methanogenic communities were enriched. A co‐culture of *Thermoanaerobacter* and *Methanothermobacter* species was obtained, pointing to a non‐obligately syntrophic glycerol degradation. This hypothesis was further studied by incubating *Thermoanaerobacter brockii* subsp. *finnii* and *T. wiegelii* with glycerol (10 mM) in pure culture and with different hydrogenotrophic methanogens. The presence of the methanogen accelerated glycerol fermentation by the two *Thermoanaerobacter* strains up to 3.3 mM day^−1^, corresponding to 12 times higher volumetric glycerol depletion rates in the methanogenic co‐cultures than in the pure bacterial cultures. The catabolic pathways of glycerol conversion were identified by genome analysis of the two *Thermoanaerobacter* strains. NADH and reduced ferredoxin formed in the pathway are linked to proton reduction, which becomes thermodynamically favourable when the hydrogen partial pressure is kept low by the hydrogenotrophic methanogenic partner.

## Graphical abstract

Glycerol is an important by‐product of the biodiesel and bioethanol industries, which results in a surplus of this compound. We investigated anaerobic glycerol fermentation coupled to methane production at high temperature (65°C), as a potential strategy for the valorization of this industrial by‐product. We discovered that glycerol fermentation by Thermoanaerobacter strains was much faster when performed in cooperative relationship with a hydrogenotrophic methanogenic partner. The methanogen facilitates glycerol conversion by consuming the hydrogen, thus assisting in the redox balance.

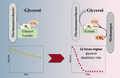

## Introduction

Worldwide demand for biodiesel increased in the last decade, leading to a global biodiesel production of 36 × 10^9^ l in 2016 (OECD/FAO, 2017). Glycerol is co‐produced in quantities that match approximately 10% of the total biodiesel production, leading to a surplus of this compound. Consequently, glycerol prices have decreased, changing glycerol from a commodity chemical to a surplus by‐product, and even a waste product (Viana *et al.*, 2012; Clomburg and Gonzalez, 2013; Ciriminna *et al.*, 2014). Glycerol is also generated in ethanol production by yeast (Navarrete *et al.*, 2014) and is frequently present in different wastes/wastewater as e.g*.* from the oleochemical industry, where waste streams can contain up to 90% glycerol (Clomburg and Gonzalez, 2013).

Anaerobic microbial processes can provide a solution for these glycerol‐rich wastes producing a wide range of valuable compounds (Viana *et al.*, 2012; Clomburg and Gonzalez, 2013). Since glycerol is a highly reduced compound, fermentative microorganisms must be able to dispose of the excess reducing equivalents, which is generally accomplished by the production of 1,3‐propanediol (1,3‐PDO), a product that is more reduced than glycerol (Clomburg and Gonzalez, 2013; Schindler *et al.*, 2014). Microorganisms that lack the 1,3‐PDO formation pathway generally transfer the electrons to hydrogen or formate, as well as to pyruvate, generating organic compounds such as ethanol, butanol or succinate (Murarka *et al.*, 2008; Scholten *et al.*, 2009; Clomburg and Gonzalez, 2013). The problem of the release of excess electrons in glycerol fermentation has been studied with diverse mesophilic bacteria, including studies on electron transfer to electrodes (Emde *et al.*, 1989; Emde and Schink, 1990) or medium sparging with inert gases for H_2_ removal (Dharmadi *et al.*, 2006; Murarka *et al.*, 2008).

The high energy content of glycerol makes it also an interesting substrate for biogas production, individually or in co‐digestion with different feedstocks – e.g. sewage sludge or the organic fraction of municipal solid wastes (Kolesárová *et al.*, 2011; Yang *et al.*, 2012). The generated biomethane may be stored or injected into the natural gas grid and used as biofuel (Beauchamp *et al.*, 2014; Hengeveld *et al.*, 2014).

The production of biodiesel and bioethanol typically generates waste streams at temperatures between 40 to 65°C. Therefore, thermophilic conditions are beneficial for valorization of glycerol. Moreover, anaerobic digestion is generally faster when performed by thermophilic than by mesophilic microorganisms (Ho *et al.*, 2013). Some thermophilic bacteria were reported to grow with glycerol in pure culture, e.g.* Thermoanaerobacter wiegelii* (Cook *et al.*, 1996), *Moorella glycerini* (Slobodkin *et al.*, 1997) and *Pseudothermotoga lettingae* (Balk *et al.*, 2002), but the thermophilic conversion of glycerol by mixed communities is only scarcely studied (Yang *et al.*, 2008; Zhang *et al.*, 2015).

This work aims to gain insight into the different microbial key players involved in glycerol degradation in mixed thermophilic anaerobic cultures. Thermophilic glycerol‐degrading cultures were enriched, and a co‐culture of *Thermoanaerobacter brockii* and a methanogenic partner was obtained, pointing to the possibility of facultatively syntrophic glycerol degradation. The influence of different methanogenic partners on glycerol degradation by two *Thermoanaerobacter* species was then investigated.

## Results

### Enrichment of glycerol‐degrading microbial cultures

**Figure 1:**
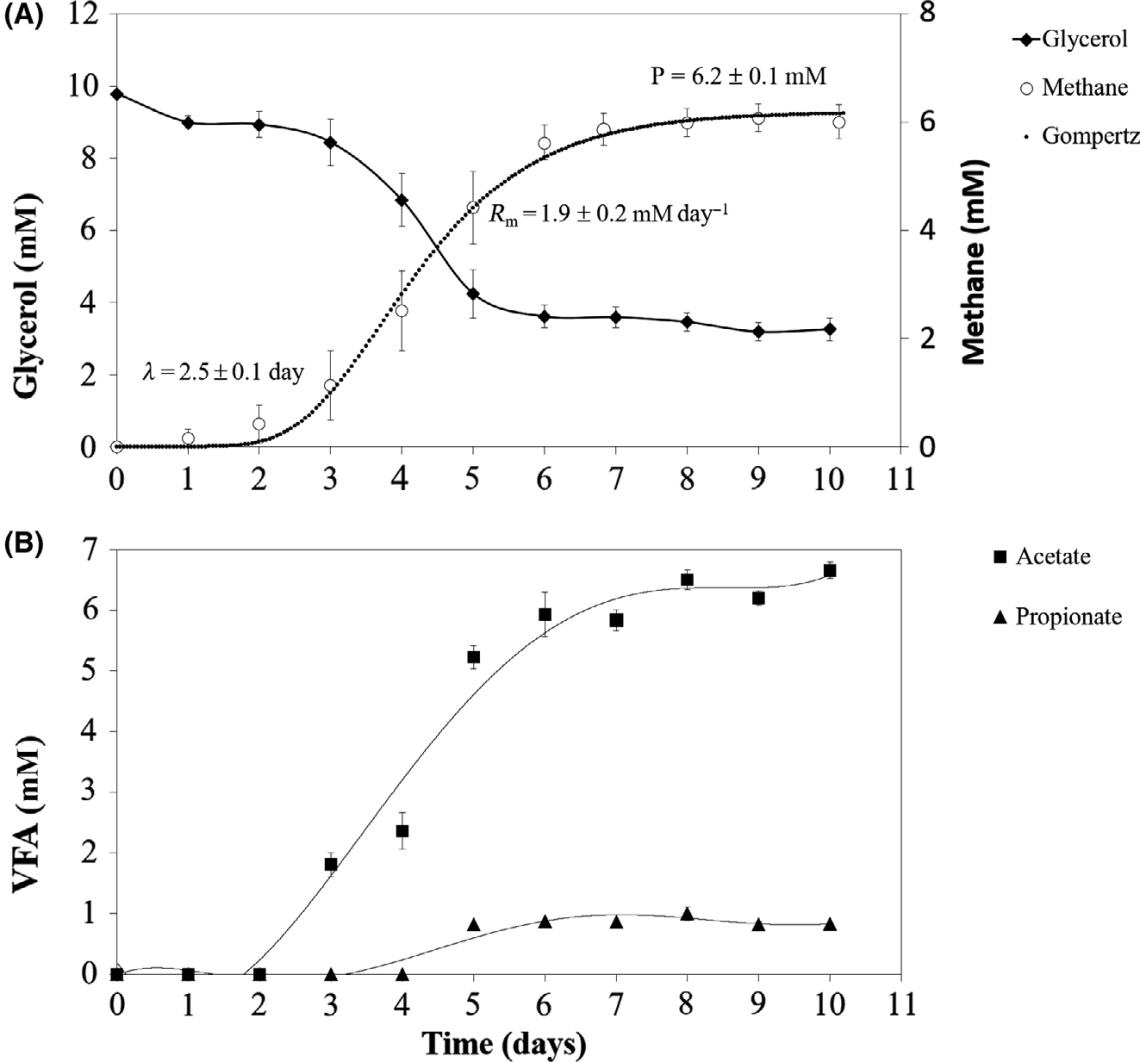
Glycerol consumption and product formation by enrichment culture Gly(9) at 55°C: glycerol concentration, experimental methane data and fitting with the modified Gompertz equation (Equation 1, *R* ^2^ = 0.984) (A); volatile fatty acids (B).

A stable thermophilic (55°C) glycerol‐degrading enrichment (culture Gly(9)) was obtained through repeated transfers to fresh medium containing glycerol as sole substrate over a period of approximately one year (Fig. S1 and Table S1). This culture converted 6.5 ± 0.3 mM of glycerol mainly to methane (6.2 ± 0.1 mM) and acetate (6.7 ± 0.1 mM) during the first 6 days of incubation (Fig. 1). Propionate was also detected, but at concentrations lower than 1 mM (Fig. 1). No other fermentation products, such as lactate, ethanol, butanol, 1,3‐PDO, 1,2‐PDO or hydrogen, were detected.

Culture Gly(9) was mainly composed by microorganisms of the genera *Methanothermobacter*, *Thermoanaerobacter*, *Pseudothermotoga* and *Acetomicrobium*, as shown in Table 1. Taxonomic identification was not possible for approximately 25% of the retrieved sequences.

**Table 1:** Microbial composition of the glycerol‐degrading enrichments Gly(9) and Col‐Gly.

Taxonomic identification^a^	Relative abundance (%)^b^				Closest relatives^c^	Identity of 16S
	Gly(9)		Col‐Gly			rRNA genes (%)^c^
*Methanothermobacter*	36.7	31.7	30.1	23.7	*Methanothermobacter wolfeii* strain SIV6 16S ribosomal RNA gene, partial sequence	100
*Thermoanaerobacter*	16.9	20.1	69.8	76.2	*Thermoanaerobacter brockii* subsp. *finnii* strain Ako‐1 16S ribosomal RNA gene, complete sequence	100
*Thermotoga*	12.0	12.6	0.0	0.0	*Pseudothermotoga profunda* AZM34c06 DNA, complete genome	98
*Acetomicrobium*	9.6	10.6	0.0	0.0	*Acetomicrobium mobile* strain NGA 16S ribosomal RNA gene, partial sequence	99
Other taxa^d^	24.9	25.1	0.0	0.0	–	–

a Taxonomic identification at the genus level based on 16S rRNA genes sequences of approximately 291 bp length by Illumina MiSeq.

b Results of duplicate samples.

c Results of sequence alignment by using BLAST towards the NCBI nucleotide database.

d Taxa with relative abundance < 1% and taxa with classification above the order level were included in *Other taxa*.

This culture was further incubated in agar‐shake cultures at 70 and 40ºC, considering that several members of the *Thermoanaerobacter* genus and all the known *Acetomicrobium* species can grow at this last temperature. A methanogenic glycerol‐degrading culture designated Col‐Gly was obtained at 40ºC, which presented very low diversity when examined by phase contrast microscopy (Fig. S2). Microbial community analysis showed the presence of only two microorganisms belonging to *Methanothermobacter* and *Thermoanaerobacter* genera, with relative abundances of 24–30% and 70–76% respectively (Table 1).

**Figure 2:**
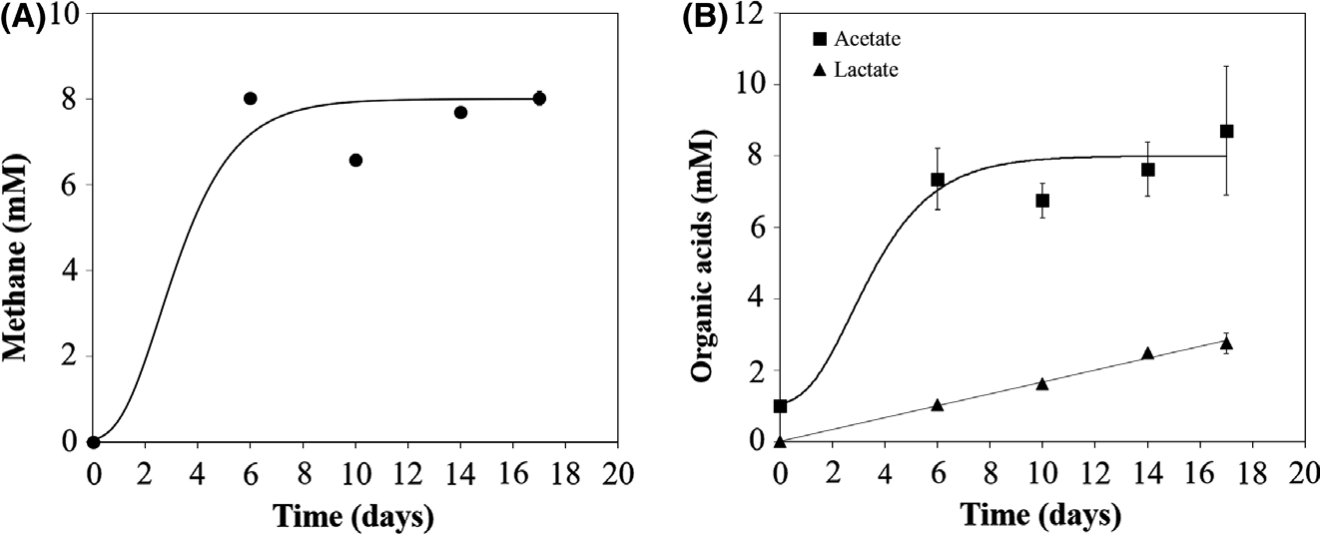
Methane (A) and organic acids (B) production by culture Col‐Gly at 65°C.

When culture Col‐Gly was incubated at 65°C (the optimal growth temperature for both identified microorganisms), 10 mM glycerol was completely degraded within 6 days of incubation (data not shown), associated with the formation of methane (8.0 ± 0.2 mM), acetate (8.7 ± 1.8 mM) and lactate (2.8 ± 0.3 mM) (Fig. 2). Hydrogen was detected at residual concentrations (< 0.01 mM) during the experiment (data not shown). Similar glycerol consumption (*i.e.* glycerol was not detectable after 7 days of incubation) and products profile (Fig. S3) were obtained in the incubations at 55°C (the original incubation temperature of the enrichment Gly(9)), and therefore further experiments were performed at 65°C.

**Figure 3:**
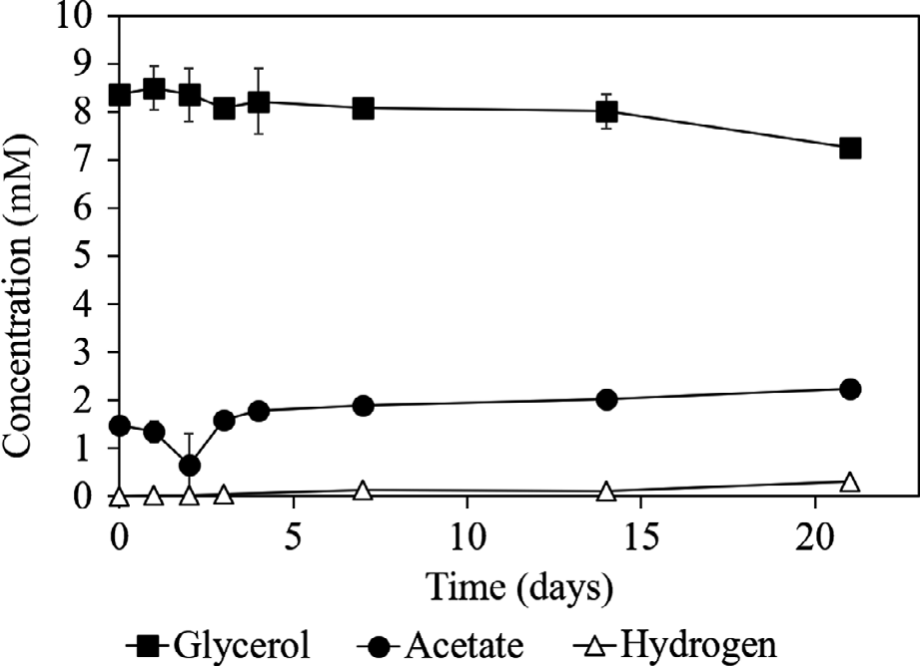
Glycerol, acetate and methane concentrations measured during the incubation of Col‐Gly with BrES.

When the enriched co‐culture Col‐Gly was incubated with BrES, a selective inhibitor of methane‐producing archaea (DiMarco *et al.*, 1990), no methane was detected in the headspace of the bottles and only vestigial glycerol consumption was observed during 7 days of incubation (Fig. 3). Hydrogen accumulated at very low amounts (< 0.1 mM), while ethanol and lactate were not detected (Fig. 3). No noteworthy effect of BrES could be detected in glycerol consumption and products formation by the *T. brockii* subsp. *finnii* type strain (Fig. S4). These results raised the hypothesis that the presence of the methanogen could influence the observed glycerol conversion rates in the enriched co‐culture Col‐Gly.

### Glycerol degradation by Thermoanaerobacter species, in pure culture or in co‐culture with methanogens

**Figure 4:**
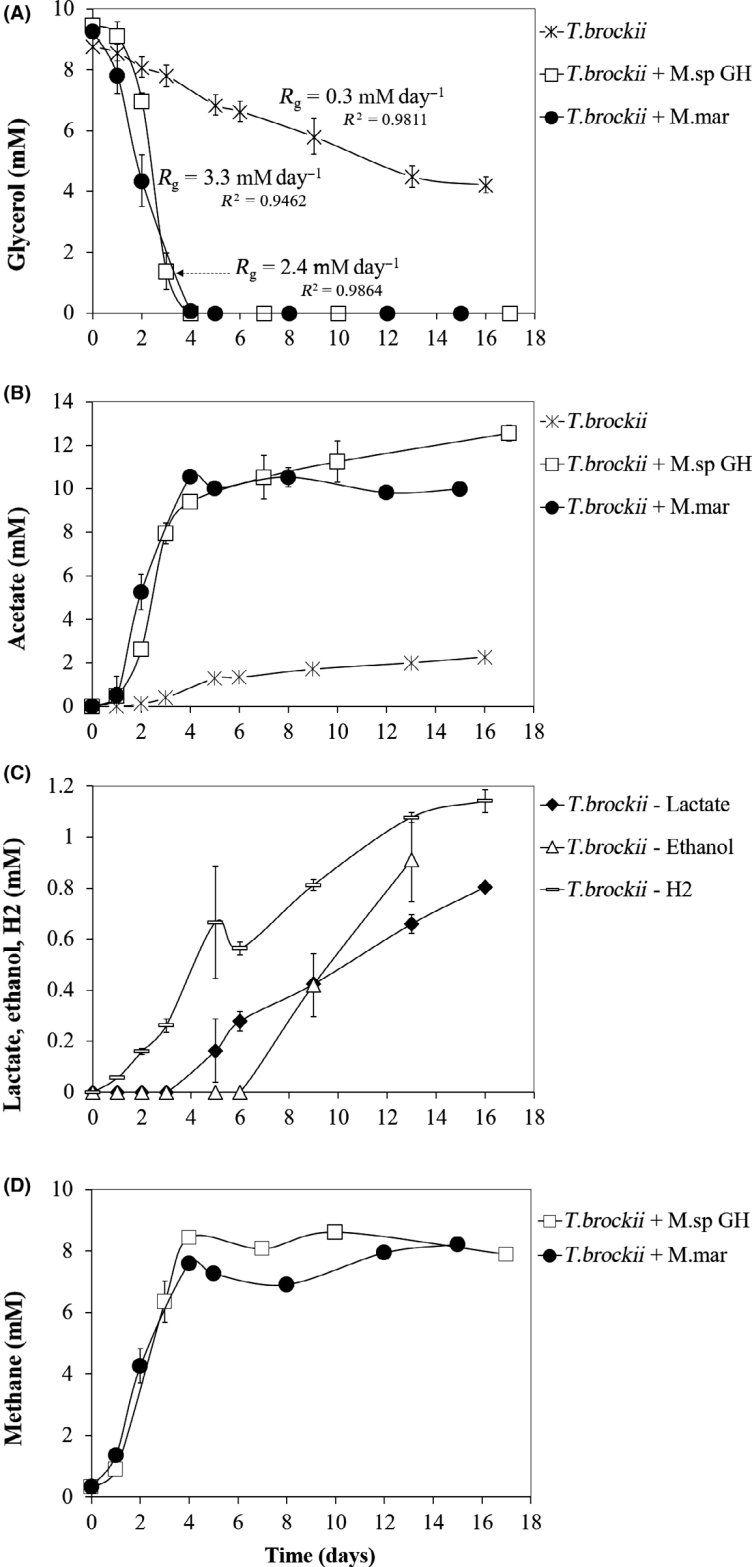
Glycerol consumption (A) and production of acetate (B) by *Thermoanaerobacter brockii* subsp. *finnii* (DSM 3389^T^) when incubated in pure culture or in co‐culture with methanogens. Lactate, ethanol and H_2_ production by *T. brockii* subsp. *finnii* in pure culture (C) and methane production in co‐culture with methanogens (D). *M.* sp. GH, culture obtained after 15 transfers of the enriched culture Col‐Gly with H_2_/CO_2_; M. mar, *Methanothermobacter marburgensis* DSM 2133^T^. *R*_g_, Volumetric glycerol depletion rate.

**Figure 5:**
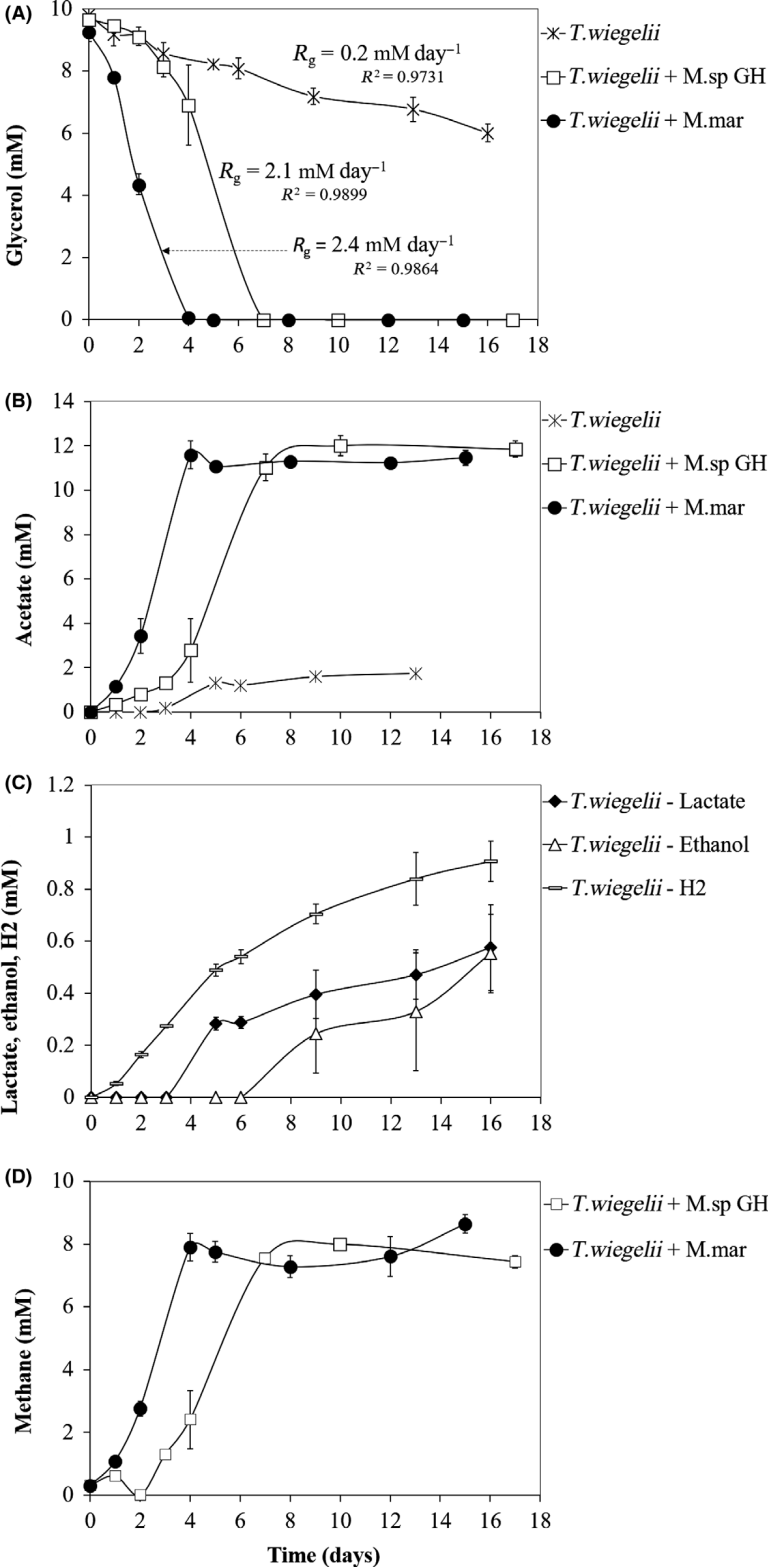
Glycerol consumption (A) and production of acetate (B) by *Thermoanaerobacter wiegelii* (DSM 10319^T^) when incubated in pure culture or in co‐culture with methanogens. Lactate, ethanol and H_2_ production by *T. wiegelii* in pure culture (C) and methane production in co‐culture with methanogens (D). *M.* sp. GH, culture obtained after 15 transfers of the enriched culture Col‐Gly with H_2_/CO_2_; M. mar, *Methanothermobacter marburgensis* DSM 2133^T^. *R*_g_, Volumetric glycerol depletion rate.

Within the *Thermoanaerobacter* genus, only *T. wiegelii*, *T. siderophilus*, *T. brockii* subsp. *finnii* and *T. subterraneus* were reported to grow with glycerol (Cook *et al.*, 1996; Slobodkin *et al.*, 1999; Fardeau *et al.*, 2000; Alves *et al.*, 2016), although growth of *T. brockii* subsp. *finnii* was described as poor (Alves *et al.*, 2016). Therefore, to further assess the possible positive effect of methanogens on glycerol fermentation, *T. brockii* subsp. *finnii* DSM 3389^T^ and *T. wiegelii* DSM 10319^T^ were grown individually in pure culture or with a methanogenic partner. *Methanothermobacter* sp. strain GH (the culture obtained after incubation of the enrichment Col‐Gly with H_2_/CO_2_ for 15 transfers) and *M. marburgensis* were the selected methanogens. The results obtained are shown in Figs 4 and 5.

After approximately 16 days of incubation, *T. brockii* subsp. *finnii* and *T. wiegelii* consumed 52 ± 4% and 39 ± 5% of the glycerol added, respectively, with corresponding volumetric substrate depletion rates (*R*_g_) of 0.3 and 0.2 mM day^−1^ (Figs 4A and 5A). Acetate was the main product (around 2 mM in both cases, Figs 4B and 5B) and hydrogen, lactate and ethanol were obtained in small amounts (0.5–1.2 mM, Figs 4C and 5C). Product yields were calculated relatively to the amount of glycerol consumed, and were similar for both species, i.e. around 0.5 mmol mmol^−1^ for acetate, 0.25 mmol mmol^−1^ for hydrogen and between 0.15 and 0.20 mmol mmol^−1^ for lactate and ethanol (Table 2). Considering the stoichiometry of the possible reactions involved (Table 3), the products measured accounted for 87% and 75% of the glycerol consumed by *T. brockii* and *T wiegelii*, respectively. When the *Thermoanaerobacter* type strains were incubated in co‐culture with the methanogens, glycerol consumption rate was substantially accelerated, i.e. all the constructed co‐cultures (*Thermoanaerobacter* strain + methanogen) completely degraded the added glycerol in 4–7 days with volumetric substrate depletion rates (*R*_g_) 8–12 times higher than the bacterial pure cultures (i.e. between 2.1 and 3.3 mM day^−1^, Figs 4A and 5A). Acetate and methane were the main products obtained (Figs 4B, D and 5B, D), with respective yield of 1.0 and 0.63–0.82 mmol mmol^−1^ relatively to the amount of glycerol consumed, which are close to the theoretically expected values (Tables 2 and 3).

**Table 2:** Product yields of glycerol fermentation, calculated relatively to the amount of glycerol consumed (mmol mmol^−1^), by *T. brockii* subsp. *finnii* (DSM 3389^T^) and *T. wiegelii* (DSM 10319^T^), when incubated in pure culture or in co‐culture with methanogens.

Culture	Acetate	Lactate	Ethanol	H_2_	Methane
*T. brockii*	0.50 ± 0.05	0.18 ± 0.01	0.20 ± 0.04	0.25 ± 0.02	n.a.
*T. brockii* + *M.* sp. GH	1.00 ± 0.04	n.d.	n.d.	n.d.	0.63 ± 0.02
*T. brockii* + *M. mar*	1.00 ± 0.00	n.d.	n.d.	n.d.	0.82 ± 0.01
*T. wiegelli*	0.46 ± 0.06	0.15 ± 0.05	0.15 ± 0.04	0.24 ± 0.04	n.a.
*T. wiegelli* + *M.* sp. GH	1.00 ± 0.04	n.d.	n.d.	n.d.	0.63 ± 0.03
*T. wiegelli* + *M. mar*	1.00 ± 0.04	n.d.	n.d.	n.d.	0.75 ± 0.03

*M.* sp. GH, culture obtained after 15 transfers of the enriched culture Col‐Gly with H_2_/CO_2_; *M. mar*, *Methanothermobacter marburgensis* DSM 2133^T^; n.a., not applicable. n.d., not determined.

**Table 3:** Possible reactions involved in glycerol degradation by the enrichment cultures Gly(9) and Col‐Gly and their corresponding Gibbs free energy changes at 25°C.

Reaction	Reactant	Main products	Equation	Δ*G* ^0’^ (kJ reaction^−1^)^a^
(1)	Glycerol	Acetate	C_3_H_8_O_3_ + 2 H_2_O → C_2_H_3_O_2_^‐^ + HCO_3_^‐^ + 3 H_2_ + 2 H^+^	−73.1
(2)	H_2_ + CO_2_	Methane	4 H_2_ + ${\text{HCO}}_{3}^{-}$ + H^+^ → CH_4_ + 3 H_2_O	−135.6
(3) = (1 + 2)	Glycerol	Acetate + Methane	C_3_H_8_O_3_ → $C_{2}H_{3}O_{2}^{-}$ + 0.75 CH_4_ + 0.25 ${\text{HCO}}_{3}^{-}$ + 0.25 H_2_O + 1.25 H^+^	−174.7
(4)	Glycerol	Lactate	C_3_H_8_O_3_ → $C_{3}H_{5}O_{3}^{-}$ + H_2_ + H^+^	−69.1
(5)	Glycerol	Ethanol	C_3_H_8_O_3_ + H_2_O → C_2_H_4_OH + ${\text{HCO}}_{3}^{-}$ + H_2_ + H^+^	−82.7

a Gibbs free energy changes (at 25°C) calculated under standard conditions (solute concentrations of 1 mM and gas partial pressure of 10^5^ Pa) at pH 7. Standard free energies of formation were obtained from Thauer *et al. *(1977) (Thauer *et al.*, 1977).

## Discussion

Glycerol (1,2,3‐propanetriol) can sustain growth of a diverse microbial community, as shown by the composition and activity of culture Gly(9) enriched at 55°C (Table 1, Fig. 1). The main bacterial genera identified were probably involved in glycerol conversion, since some of the characterized strains within the genera *Pseudothermotoga*, *Acetomicrobium* and *Thermoanaerobacter* have been reported as glycerol degraders (Rees *et al.*, 1997; Menes and Muxí, 2002; Maru *et al.*, 2013; Alves *et al.*, 2016)*.* Acetate and methane were the main products of glycerol conversion, indicating that methane was produced from formate or H_2_/CO_2_ (Fig. 1). This was reinforced by the identification of *Methanothermobacter* sp., a hydrogenotrophic methanogen, as the only archaeon in this community (Table 1).

When applying a lower temperature (40ºC) as selective factor, a co‐culture composed by *Thermoanaerobacter* and *Methanothermobacter* was enriched (culture Col‐Gly). This co‐culture was capable of fast glycerol degradation (< 6 days) coupled to good growth evaluated by visual inspection. Also in the work of Zhang *et al. *(2015), *Thermoanaerobacter* spp. and hydrogenotrophic methanogens (mainly *Methanothermobacter thermautotrophicus*) were the dominant microorganisms in the community developed in a continuous bioreactor operated with glycerol at 70°C. However, as previously mentioned, Alves *et al. *(2016) reported that glycerol was only poorly utilized by *T. brockii* subsp. *finnii*. When we incubated pure cultures of *T. brockii* subsp. *finnii* or *T. wiegelii* with glycerol at 10 mM, glycerol was hardly fermented, *i.e.* more than 16 days were required to convert 40–50% of the added glycerol (Figs 4 and 5). In spite of that, these two strains easily ferment glucose in pure culture, e.g*.* Alves *et al. *(2016) reported degradation of 20 mM of glucose by *T. brockii* subsp. *finnii* and *T. wiegelli* in 7 and 3 days respectively. This difference is probably related to the more reduced nature of glycerol, which leads to the generation of twice the number of reducing equivalents per pyruvate molecule formed, compared with glucose (Clomburg and Gonzalez, 2013).

**Figure 6:**
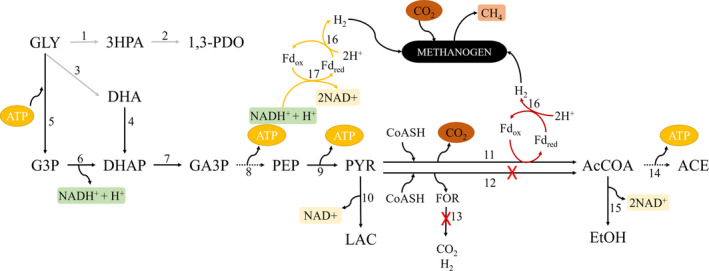
Main metabolic pathway for glycerol conversion by *Thermoanaerobacter brockii* subsp. *finnii* (DSM 3389^T^) and *Thermoanaerobacter wiegelii* (DSM 10319^T^). Grey lines: absent in *Thermoanaerobacter*. Broken lines mean multiple steps. Abbreviations: GLY, glycerol; 3HPA, 3‐hydroxypropionaldehyde; 1,3‐PDO, 1,3‐propanediol; DHA, dihydroxyacetone; DHAP, dihydroxyacetone phosphate; G3P, glycerol‐3‐phosphate; GA3P, glyceraldehyde‐3‐phosphate; PEP, phosphoenolpyruvate; PYR, pyruvate; LAC, lactate; AcCOA, acetyl‐coenzyme A; FOR, formate; ACE, acetate; EtOH, ethanol; Fd_ox_, oxidized ferredoxin; Fd_red_, reduced ferredoxin. 1, glycerol dehydratase; 2, 1,3‐PDO dehydrogenase; 3, glycerol dehydrogenase; 4, dihydroxyacetone kinase; 5, glycerol kinase; 6, glycerol‐3‐phosphate dehydrogenase; 7, triosephosphate isomerase; 8, glyceraldehyde‐3‐phosphate dehydrogenase, phosphoglycerate kinase, phosphoglycerate mutase and enolase; 9, pyruvate kinase; 10, L‐lactate dehydrogenase; 11, pyruvate:ferredoxin oxidoreductase; 12, pyruvate formate lyase; 13, formate hydrogen lyase; 14, phosphate acetyltransferase and acetate kinase; 15, acetaldehyde dehydrogenase; 16, hydrogenase; and 17, ferredoxin‐NADP(+) reductase. Additional information of EC number and genome location of the enzymes of this metabolic pathway can be found at Table S2.

The two *Thermoanaerobacter* strains studied in this work did not produce 1,3‐PDO, and the analysis of their genomes confirmed that these bacteria lack the genes encoding for the enzymes involved in the pathway of 1,3‐PDO formation (enzymes 1 and 2, Fig. 6). Therefore, these bacteria are not able to easily dispose of the excess of reducing equivalents generated from glycerol; as lactate and ethanol (+CO_2_) are more oxidized than glycerol, formation of these compounds cannot balance the surplus of electrons formed in the conversion of glycerol to acetate, and hydrogen production is constrained by thermodynamics (Figs 4, 5, 6, Table 3). The two *Thermoanaerobacter* strains oxidize and decarboxylate pyruvate to acetyl‐coenzyme A with ferredoxin as redox mediator, as indicated by the presence in their genomes of the genes coding for pyruvate:ferredoxin oxidoreductase (enzyme 11, Fig. 6), and by the absence of pyruvate formate lyase and formate hydrogen lyase encoding genes (Fig. 6 – enzymes 12 and 13 respectively). Subsequently, a trimeric or a monomeric hydrogenase (enzyme 16) oxidizes ferredoxin and, using protons as final electron acceptors, leads to hydrogen production. In the reoxidation of NADH^+^ + H^+^, electrons are transferred by a NADH:ferredoxin oxidoreductase (enzyme 17) to a hydrogenase (trimeric or monomeric, enzyme 16) and thus hydrogen can also be produced (Vardar‐Schara *et al.*, 2006; Calusinska *et al.*, 2010).

The oxidation of reduced ferredoxin and especially NADH coupled to proton reduction only becomes thermodynamically feasible at low hydrogen partial pressure (Sousa *et al.*, 2009; Stams and Plugge, 2009). Therefore, *Thermoanaerobacter* strains surpass the metabolic dilemma of redox balancing and energy acquisition when in the presence of a methanogen, which consumes the hydrogen produced during glycerol fermentation and functions as biological electron acceptor (Fig. 6). In fact, glycerol conversion to acetate becomes more exergonic and thermodynamically more favourable if the hydrogen produced is used by hydrogenotrophic methanogens to produce methane, as shown by the Gibbs free energy changes of Δ*G* ^0^’ = −73.1 and −174.7 kJ reaction^−1^ respectively (reactions 1 and 3, Table 3). In these co‐cultures, ethanol production could not be detected, and only small amounts of lactate were produced by the enriched culture Col‐Gly (Figs 4 and 5).

The positive effect of the methanogen was also experimentally confirmed when the methanogenic activity in culture Col‐Gly was inhibited by BrES, which caused glycerol degradation to proceed at a much lower rate (Fig. 3). The relationship between the *Thermoanaerobacter* strains and the methanogen points to a facultative syntrophy, since glycerol fermentation can be performed by these bacteria in pure culture but their growth and metabolic products are directly influenced by the hydrogen scavenger (Stams and Plugge, 2009). This syntrophic relationship is energetically advantageous for the *Thermoanaerobacter* bacteria, compared with glycerol fermentation in pure culture, since it allows higher ATP gain, i.e. 2 ATP are formed from glycerol to acetate instead of 1 from glycerol to lactate and/or ethanol (Fig. 6). Since syntrophic glycerol fermentation by the two *Thermoanaerobacter* strains does not involve a pyruvate formate lyase, the ability of the methanogenic partner to consume formate is not needed for this interspecies relationship. Only a slight delay of approximately 2 days was observed in the incubations of *T. wiegellii* and the *Methanothermobacter.* sp. strain GH (Fig. 5).

The importance of an external electron acceptor for improving glycerol conversion has been previously reported, for example in cultures of *Actinobacillus succinogenes* grown in the presence of dimethylsulfoxide (DMSO) as external electron acceptor (Carvalho *et al.*, 2014; Schindler *et al.*, 2014). Moreover, glycerol fermentation by *Escherichia coli* was shown to be impaired by hydrogen accumulation (Dharmadi *et al.*, 2006; Gonzalez *et al.*, 2008), which could be overcome by co‐cultivation with the methanogen *Methanobacterium formicicum* (Richter and Gescher, 2014; Kim *et al.*, 2017). Likewise, glycerol fermentation by *E. coli* and *Propionibacterium freudenreichii* can be supported through electron transfer to electrodes mediated by potassium ferricyanide (Emde *et al.*, 1989; Emde and Schink, 1990). For *Thermoanaerobacter brockii* subsp. *brockii*, the addition of thiosulphate or *Methanobacterium* sp. as electron acceptors improved the oxidative deamination of aminoacids (Fardeau *et al.*, 1997). The consumption of glucose and pyruvate by *Thermoanaerobium brockii* was enhanced as well by using acetone as electron acceptor (Ben‐Bassat *et al.*, 1981). Also, Vipotnik *et al. *(2016) showed inhibition of glucose consumption by *Thermoanaerobacter* strain AK68 when exposed to high hydrogen partial pressure and that the addition of thiosulphate or co‐cultivation with *Methanothermobacter* strain M39 (as electron scavenger) increased the utilization of glucose and acetate production (Vipotnik *et al.*, 2016). In summary, we show that the presence of a hydrogenotrophic methanogenic partner, acting as biological electron acceptor, enhances glycerol conversion by *Thermoanaerobacter* species, since it facilitates the redox balance and contributes to a higher energy gain by these bacteria. Therefore, syntrophic glycerol fermentation promotes faster anaerobic treatment of glycerol‐rich waste streams coupled to methane production.

## Experimental procedures

### Biomass source

Thermophilic anaerobic sludge was collected from a lab‐scale up‐flow anaerobic column reactor operated at 55°C, fed with a mixture of skim milk and sodium oleate (50:50% of the chemical oxygen demand, COD) at a COD concentration of 10 g l^−1^ and hydraulic retention time of 1 day. Additional details of the reactor operation are provided in Supporting Information. Degradation of the substrate accumulated during the reactor operation was promoted by incubation in batch at 55°C for 18 days, before starting the enrichments.

### Medium composition and cultivation

All the experiments were performed using a bicarbonate‐buffered mineral salt medium (basal medium, BM) prepared as described by Stams *et al. *(1993). BM was dispensed in serum bottles which were sealed with butyl rubber septa and aluminum crimp caps. The headspace of the bottles was flushed with a gas mixture of N_2_ and CO_2_ (80:20% v/v), at a final pressure of 1.7 × 10^5^ Pa. Before incubation, the medium was reduced with 0.8 mM sodium sulfide and supplemented with salts and vitamins. All inoculations and transfers were done aseptically using sterile syringes and needles.

### Enrichment of glycerol‐degrading microbial cultures

Enrichments (coded Gly(x), where × represents the number of transfers) were started by inoculating 120 ml serum bottles, containing 50 ml BM, with 10% (v/v) of the sludge. Glycerol was added from a sterile stock solution to a final concentration of 10 mM, based on the works of Fardeau *et al. *(2000) and Alves *et al. *(2016). Successive transfers of the cultures to new medium (10% v/v) and serial dilutions were made after confirming microbial growth and activity, based on microscopic observations and methane measurements (more than 30% of the theoretical value expected). All cultures were incubated at 55°C, statically and in the dark. Schematic representation of the experimental procedure applied is shown in Fig. S1.

Physiological characterization was performed after nine successive transfers (enrichment Gly(9)), in triplicate 500 ml bottles containing 250 ml BM (Fig. S1) and glycerol (10 mM). Methane content in the headspace, volatile fatty acids (VFA), lactate, glycerol, ethanol, butanol, 1,3‐PDO and 1,2‐PDO were measured daily. The final hydrogen content of the headspace was also measured. In addition, duplicate samples were collected at the end of the incubation period for DNA extraction and 16S rRNA genes sequencing by Illumina MiSeq. The experimental methane production data was fitted by the modified Gompertz equation (equation 1) for estimation of the methane production kinetics (Zwietering *et al.*, 1990).

(1) $$M\left(t\right)=P\times \exp\left\{-\exp\left[\frac{R_{m}\cdot e}{P}\left(\lambda -t\right)+1\right]\right\},$$

where *M (t)* = cumulative methane production (mM), *P = *maximum methane production (mM), *R_m_* = methane production rate (mM day^−1^), *e* = 2.7182818 and λ = lag phase (days). The standard error for each variable and the coefficient of determination (*R* ^2^) were calculated.

Further microbial selection was then performed by serially diluting the enrichment Gly(9) in agar‐shake cultures, containing 50 ml BM solidified with 1.5% (w/v) agar. Incubations were made at 40 and 70°C, in the dark and without agitation. Colonies were picked and transferred to the same medium without agar. Growth (verified by visual inspection of the bottles and by microscopic observations) was only observed in the cultures incubated at 40°C, and thus the enrichments at 70°C were not continued. At 40°C, after four successive transfers in liquid medium, microbial community composition was analysed by sequencing of 16S rRNA genes (Illumina MiSeq). This culture was coded Col‐Gly. Its ability to consume glycerol (10 mM) and the products formed (methane, hydrogen, VFA, lactate, alcohols) were monitored in triplicate assays, incubated at two different temperatures (55 and 65°C). Incubation with glycerol in the presence of 20 mM of 2‐bromoethanesulfonate (BrES) was also performed in triplicate, and glycerol, acetate, methane and hydrogen concentrations were measured with time.

### Glycerol degradation by *Thermoanaerobacter* species, in pure culture or in co‐culture with methanogens

*Thermoanaerobacter brockii* subsp. *finnii* (DSM 3389^T^), *Thermoanaerobacter wiegelii* (DSM 10319^T^) and *Methanothermobacter marburgensis* (DSM 2133^T^) were obtained from the Deutsche Sammlung von Mikroorganismen und Zellkulturen (DSMZ, Braunschweig, Germany). The two *Thermoanaerobacter* strains were grown with 20 mM glucose, and *M. marburgensis* was cultured using a gas phase of H_2_/CO_2_ (80:20% v/v, 1.7 × 10^5^ Pa). Enrichment Col‐Gly was also incubated with H_2_/CO_2_ (80:20% v/v, 1.7 × 10^5^ Pa) for 15 transfers, with the aim of retrieving the methanogen from this culture. The culture obtained was named *Methanothermobacter* sp. strain GH.

An assay was then set up to study glycerol degradation by the two *Thermoanaerobacter* species, in pure culture or in co‐culture with methanogens. For this, the two cultures of methanogens (*Methanothermobacter* sp. strain GH and *M. marburgensis*) were pre‐grown until complete hydrogen consumption, after which the headspace of the bottles was flushed and pressurized with N_2_/CO_2_ (80:20% v/v, 1.7 × 10^5^ Pa) under sterile conditions. The two *Thermoanaerobacter* type strains were also pre‐grown with glycerol and were then transferred (10% v/v) to bottles containing fresh medium or mixed with the pre‐grown cultures of methanogens. Glycerol was added at 10 mM. The concentrations of glycerol, other alcohols, VFA, lactate, methane and hydrogen were measured over time. All tests were performed in triplicate. Incubations were made at 65°C, statically and in the dark.

### Analytical methods

Phase contrast microscopy was performed using an Olympus CX41 RF microscope, and micrographs were obtained with an Olympus Altra 20 image acquisition system. The software used with this setup was the AnalySIS getIT (Olympus soft imaging solutions GmbH). Methane and hydrogen were quantified by gas chromatography. For methane quantification, a GC‐2014 Shimadzu gas chromatograph was used with a Porapak Q column and a flame ionization detector. N_2_ was used as carrier gas. Injection port, column and detector temperatures were 110, 35 and 220°C respectively. Hydrogen was analysed using a Molsieve column (MS‐13x 80/100 mesh) and a thermal conductivity detector Bruker Scion 456 Chromatograph (Bruker, Billerica, MA, USA) with argon (60 ml min^−1^) as the carrier gas. The injector, column and detector temperatures were 100, 35 and 130°C respectively. Volatile fatty acids (VFA), lactate, glycerol and other alcohols were analysed by high‐performance liquid chromatography (HPLC; Jasco, Tokyo, Japan). For organic acids quantification, an Agilent Hi‐Plex H (300 × 7.7 mm) column was used, with a mobile phase of 2.5 mM H_2_SO_4_ at a flow rate of 0.6 ml min^−1^. The column temperature was set at 60°C and spectrophotometric ultraviolet (UV) detection was performed at 210 nm. Glycerol and other alcohols were analysed using a Varian Aminex 87H (300 × 7.8 mm) with a mobile phase of 5 mM H_2_SO_4_ at a flow rate of 0.7 ml min^−1^, with the column temperature set at 60°C and refractive index (RI) detection.

### Microbial composition of the glycerol‐degrading enrichment cultures

Aliquots of well‐homogenized sludge were collected from cultures Gly(9) and Col‐Gly, and immediately frozen at −20°C. Total genomic DNA was extracted using the FastDNA SPIN Kit for Soil (MP Biomedicals, Solon, OH) and purified by ethanol precipitation. DNA amplification, Illumina library preparation, amplicon sequencing (Illumina MiSeq, Inc. San Diego, CA, USA) and bioinformatics analysis of the data were performed at Research and Testing Laboratory (Lubbock, TX, USA). Samples were amplified for sequencing using the universal primer pair 515f and 806r (Caporaso *et al.*, 2011), targeting the prokaryotic 16S rRNA gene. Details on the sequencing and bioinformatics data analysis can be found elsewhere (Paulo *et al.*, 2017). All sequencing reads were submitted to the European Nucleotide Archive (ENA) under the study accession number PRJEB30535 (http://www.ebi.ac.uk/ena/data/view/PRJEB30535). A comparison of 16S rRNA gene sequences of OTU to the NCBI database was also performed using the BLASTN alignment tool (http://ncbi.nlm.nih.gov/blast).

### Genome analysis of Thermoanaerobacter strains

Analysis of *Thermoanaerobacter brockii* subsp. *finnii* (DSM 3389^T^) and *Thermoanaerobacter wiegelii* (DSM 10319^T^) genomes, was performed using the Integrated Microbial Genomes (IMG) (https://img.jgi.doe.gov/) and The National Center for Biotechnology Information (NCBI) (https://www.ncbi.nlm.nih.gov/) genomic platforms.

## Conflict of interest

None declared.

## Supporting information


**Figure S1** **.** Experimental procedure applied for the enrichment of thermophilic glycerol‐degrading microbial cultures at 55ºC.
**Figure S2** **.** Phase contrast micrograph of culture Col‐Gly.
**Figure S3** **.** Methane (A) and organic acids (B) production by enriched culture Col‐Gly at 55°C.
**Figure S4** **.** Glycerol consumption (A) and production of acetate (B) and H_2_ (C) by *T. brockii* subsp. *finnii *type strain, when incubated with BrES (w/BrES) and without BrES (w/o BrES).
**Table S1** **.** Methane production from glycerol by the different generations (coded Gly(x), where × represents the number of transfers), during the enrichment process.
**Table S2** **.** Additional information about the enzymes involved in the metabolic pathway for glycerol conversion of *Thermoanaerobacter brockii* subsp. *finnii* (DSM 3389^T^) and *Thermoanaerobacter wiegelii* (DSM 10319^T^). The data were retrieved from NCBI genomic platform.
