# Supplementary material for: Co‐cultivation of Thermoanaerobacter strains with a methanogenic partner enhances glycerol conversion
Source: Microb Biotechnol. 2020 Mar 10;13(4):962–73. doi: 10.1111/1751-7915.13506 (PMC7264899; doi:10.1111/1751-7915.13506)
Supplement: Supplementary file 1 — Figure S1 . Experimental procedure applied for the enrichment of thermophilic glycerol‐degrading microbial cultures at 55ºC. Figure S2 . Phase contrast micrograph of culture Col‐Gly. Figure S3 . Methane (A) and organic acids (B) production by enriched culture Col‐Gly at 55°C. Figure S4 . Glycerol consumption (A) and production of acetate (B) and H2 (C) by T. brockii subsp. finnii type strain, when incubated with BrES (w/BrES) and without BrES (w/o BrES). Table S1 . Methane production from glycerol by the different generations (coded Gly(x), where × represents the number of transfers), during the enrichment process. Table S2 . Additional information about the enzymes involved in the metabolic pathway for glycerol conversion of Thermoanaerobacter brockii subsp. finnii (DSM 3389T) and Thermoanaerobacter wiegelii (DSM 10319T). The data were retrieved from NCBI genomic platform. [file MBT2-13-962-s001.docx]

# Cocultivation of *Thermoanaerobacter* strains with a methanogenic partner enhances glycerol conversion

Magalhães CP,^a^ Ribeiro JA,^a^^[[1]](#footnote-1)^ Guedes AP,^a¥^^[[2]](#footnote-2)^ Arantes AL,^a^ Sousa DZ,^a,b^ Stams AJM,^a,b^ Alves, MM,^a^ Cavaleiro AJ^a#^

^a^ Centre of Biological Engineering, University of Minho, Braga, Portugal

^b^ Laboratory of Microbiology, Wageningen University & Research, Wageningen, The Netherlands

# Corresponding Author: Ana Júlia Cavaleiro, [acavaleiro@deb.uminho.pt](mailto:acavaleiro@deb.uminho.pt)

Centre of Biological Engineering, University of Minho, Campus de Gualtar, 4710-507 Braga, Portugal. Tel.: +351253604423; Fax.: +351253604429.

Running title: Enhanced glycerol conversion by *Thermoanaerobacter*

**Contents:**

**Supporting Methods**

Biomass source

**Supporting Tables**

**Table S1.** Methane production from glycerol by the different generations (coded Gly(x), where x represents the number of transfers), during the enrichment process.

**Table S2.** Additional information about the enzymes involved in the metabolic pathway for glycerol conversion of *Thermoanaerobacter brockii* subsp. *finnii* (DSM 3389^T^) and *Thermoanaerobacter wiegelii* (DSM 10319^T^). The data were retrieved from NCBI genomic platform.

**Supporting Figures**

**Figure S1.** Experimental procedure applied for the enrichment of thermophilic glycerol-degrading microbial cultures at 55 °C.

**Figure S2.** Phase contrast micrograph of culture Col-Gly.

**Figure S3.** Methane (a) and organic acids (b) production by enriched culture Col-Gly at 55 °C.

**Figure S4.** Glycerol consumption (a) and production of acetate (b) and H_2_ (c) by *T. brockii* subsp. *finnii* type strain, when incubated with BrES (w/BrES) and without BrES (w/o BrES).

**Supporting Methods**

***Biomass source***

Thermophilic anaerobic sludge was collected from a lab-scale up-flow anaerobic column reactor operated at 55 °C. The reactor was initially inoculated with mesophilic granular sludge from a brewery wastewater treatment plant (Sociedade Central de Cervejas e Bebidas, S.A., Portugal). Adaptation to thermophilic conditions was promoted during 170 days of operation, through which the temperature was increased from 37 °C to 55 °C, at 5 °C per week. From day 170 until the end of the operation (284 days) the temperature was set at 55 °C, and the reactor was fed with a mixture of skim milk and sodium oleate (50:50 % of the chemical oxygen demand, COD), supplemented with macronutrients, micronutrients and bicarbonate, as previously described by Alves *et al.* 2001. The sludge used as inoculum for enriching glycerol-degrading bacteria was collected from the reactor at day 255 of operation, after a period of reactor loading with 10 g L^-1^ day^-1^, expressed as COD, and hydraulic retention time of 1 day. Degradation of the substrate accumulated during the reactor operation was promoted by incubation in batch at 55 °C for 18 days, before starting the enrichments.

1. Alves MM, Mota Vieira JA, Álvares Pereira RM, Pereira MA, Mota M. 2001. Effects of lipids and oleic acid on biomass development in anaerobic fixed-bed reactors. Part II: Oleic acid toxicity and biodegradability. Water Res 35:264–270.

**Table S1.** Methane production from glycerol by the different generations (coded Gly(x), where x represents the number of transfers), during the enrichment process.

| **Culture** | **Time (days)** | **CH_4_ (mmol L^-1^)** | |
| --- | --- | --- | --- |
| Gly(3) | 11 | 6.1 |  |
| Gly(4) | 25 | 6.5 |  |
| Gly(5) | 14 | 7.2 |  |
| Gly(6) | 27 | 7.0 |  |
| Gly(7) | 10 | 6.0 |  |

**Table S2.** Additional information about the enzymes involved in the metabolic pathway for glycerol conversion of *Thermoanaerobacter brockii* subsp. finnii (DSM 3389^T^) and *Thermoanaerobacter wiegelii* (DSM 10319^T^). The data were retrieved from NCBI genomic platform. _, Not present

| **Enzyme nº** | **Enzyme** | **EC number** | **Locus Tag**  ***T. brockii*** | **Locus Tag**  ***T. wiegelii*** |
| --- | --- | --- | --- | --- |
| 1 | glycerol dehydratase | 4.2.3.30 | _ | _ |
| 2 | 1,3-propanediol dehydrogenase | 1.1.1.202 | _ | _ |
| 3 | glycerol dehydrogenase | 1.1.1.6 | _ | _ |
| 4 | dihydroxyacetone kinase | 2.7.1.121 | Thebr_0556 - 0558 | Thewi_2002 |
| 5 | glycerol kinase | 2.7.1.30 | Thebr_0551 | Thewi_2009 |
| 6 | glycerol 3-phosphate dehydrogenase | 1.1.1.94 | Thebr_0863 | Thewi_1575 |
| 7 | triosephosphate isomerase | 5.3.1.1 | Thebr_0755 | Thewi_1687 |
| 8.1 | glyceraldehyde-3-phosphate dehydrogenase | 1.2.1.12 | Thebr_0753 | Thewi_1689 |
| 8.2 | phosphoglycerate kinase | 2.7.2.3 | Thebr_0754 | Thewi_1688 |

**Table 2.** (continuation)

| **Enzyme nº** | **Enzyme** | **EC number** | **Locus Tag**  ***T. brockii*** | **Locus Tag**  ***T. wiegelii*** |
| --- | --- | --- | --- | --- |
| 8.3 | phosphoglycerate mutase | 5.4.2.1 | Thebr_0756 | Thewi_2490 |
| 8.4 | enolase | 4.2.1.11 | Thebr_0757 | Thewi_1685 |
| 9 | pyruvate kinase | 2.7.1.40 | Thebr_0702 | Thewi_1786 |
| 10 | l-lactate dehydrogenase | 1.1.1.27 | Thebr_2046 | Thewi_0277 |
| 11 | pyruvate:ferredoxin oxidoreductase | 1.2.7.1 | Thebr_0298 | Thewi_0500 |
| 12 | pyruvate formate-lyase | 2.3.1.54 | _ | _ |
| 13 | formate hydrogen-lyase | 1.17.1.9 | _ | _ |
| 14.1 | phosphate acetyltransferase | 2.3.1.8 | Thebr_1325 | Thewi_1436 |
| 14.2 | Acetate kinase | 2.7.2.1 | Thebr_1324 | Thewi_1435 |
| 15 | Acetaldehyde dehydrogenase | 1.2.1.10 | Thebr_0212 | Thewi_2535 |
| 16 | ferredoxin-NADP(+) redutase | 1.18.1.2 | Thebr_0223 | Thewi_2519 |
| 17 | Hydrogenase | 1.12.1.3 | Thebr_1491 | Thewi_0980 |


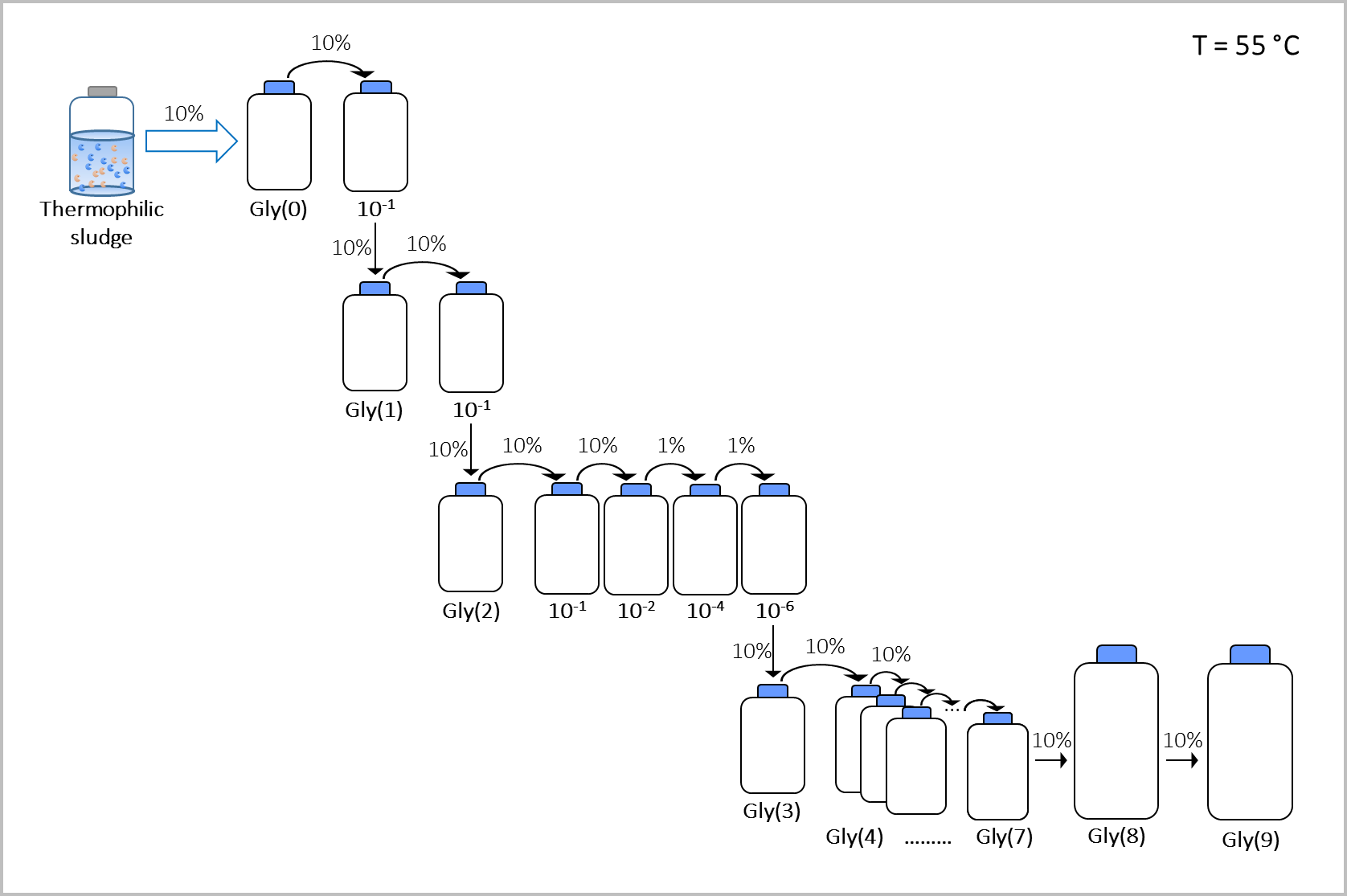


**Figure S1.** Experimental procedure applied for the enrichment of thermophilic glycerol-degrading microbial cultures at 55 °C.


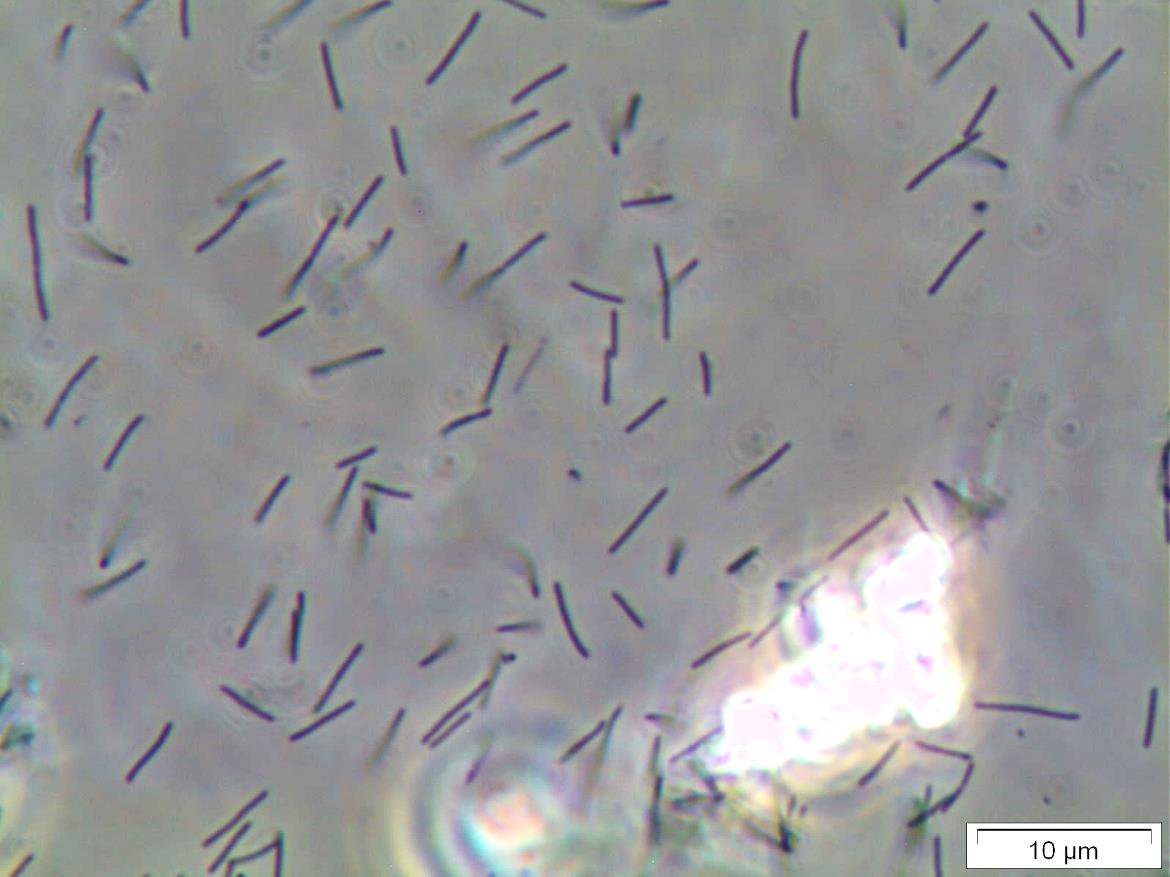

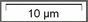


**Figure S2.** Phase contrast micrograph of culture Col-Gly.


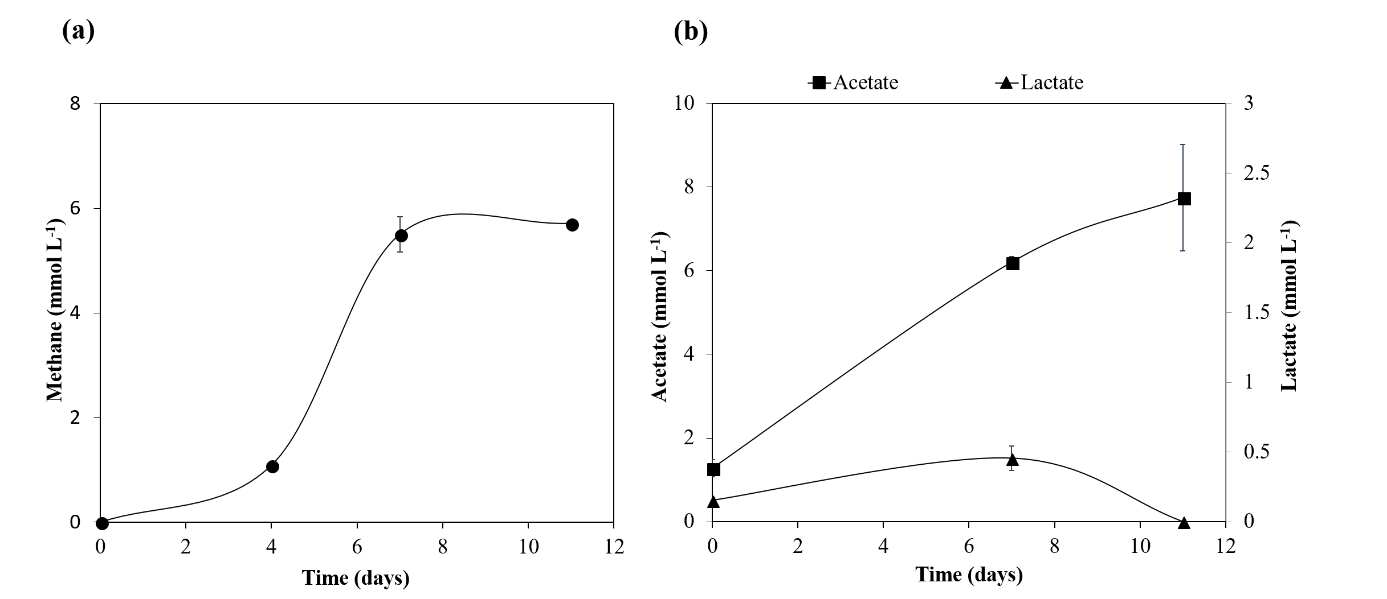


**Figure S3.** Methane (a) and organic acids (b) production by enriched culture Col-Gly at 55 °C.


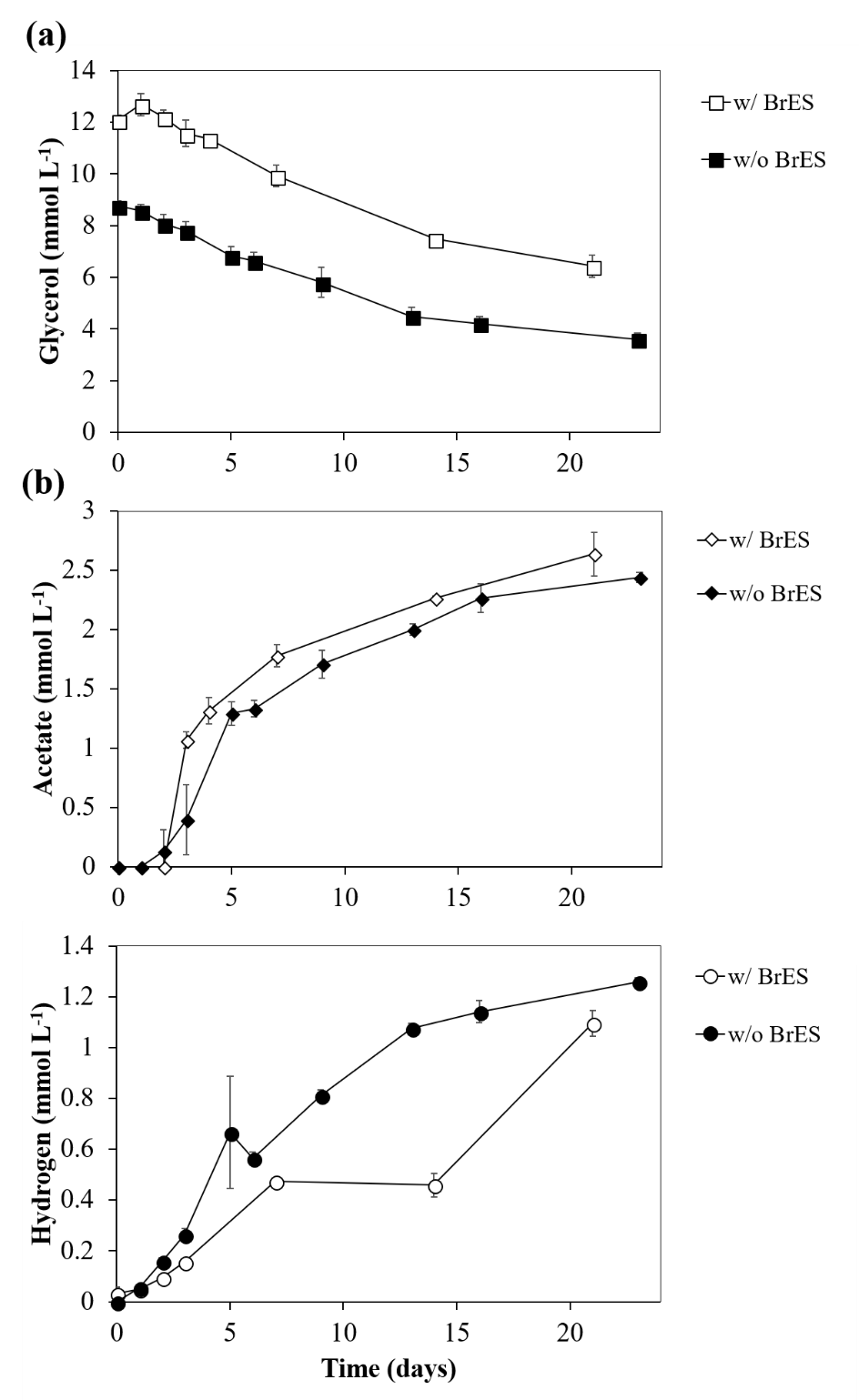


**(c)**

**Figure S4.** Glycerol consumption (a) and production of acetate (b) and H_2_ (c) by *T. brockii* subsp. *finnii* type strain, when incubated with BrES (w/ BrES) and without BrES (w/o BrES).

1. Present address: JA Ribeiro, Optimizer - Serviços e Consultadoria Informática Lda., Porto, Portugal. [↑](#footnote-ref-1)
2. Present address: AP Guedes, Agricultural Superior School of Ponte de Lima, Polytechnic Institute of Viana do Castelo, Portugal. [↑](#footnote-ref-2)
